# Supplementary material for: A Perfect Plastic Material for Studies on Self-Propelled Motion on the Water Surface
Source: Molecules. 2021 May 23;26(11):3116. doi: 10.3390/molecules26113116 (PMC8197156; doi:10.3390/molecules26113116)
Supplement: Supplementary file 1 [file molecules-26-03116-s001.zip › Loffler-et-al-Supplementary-Information.pdf]

A Perfect Plastic Material for Studies on Self-Propelled Motion on the Water Surface – Supplementary Information

Richard J.G. Loffler,[a] Martin M. Hanczyc,[b‡] and Jerzy Gorecki,[*a]
[a] Institute of Physical Chemistry, Polish Academy of Sciences,
Kasprzaka 44/52, Warsaw 01-224, Poland
E-mail: jgorecki@ichf.edu.pl
[b] Laboratory for Artificial Biology, Centre for Integrative Biology (CIBIO),
University of Trento, Polo Scientifico e Tecnologico Fabio Ferrari, Polo B,
Via Sommarive 9, 38123 Povo, TN, Italy
[c]Chemical and Biological Engineering, University of New Mexico,
Albuquerque, NM, USA

March 13, 2021

SI.1 The motion of self-propelled pill for different weight ratios of camphor, camphene, and polypropylene

In the Supplementary Information, we present the analysis of the motion of a pill made of camphor, camphene, and polypropylene inside a Petri dish with diameters of 11 cm or 12 cm. In experiments, we considered different weight fractions of camphor, camphene, and polypropylene. We analyze the shape of the trajectory, the distribution of distances between the pill and the dish center, the pill speeds as functions of time, and the probability distributions of the observed values of speed. For each ratio of components, we present the results of three independent experiments. Results coming from a single experiment can be distinguished by the color of curves used to plot them.

As for the camphene-polypropylene plastic system (see Fig 1 in the paper), we observed an initial decrease in the pill speed. For the camphene-polypropylene plastic, the speed of a pill stabilized after 30 s. In the case of camphene-camphor-polypropylene plastic, the speed stabilization is much slower, and it can be observed after 300 s as illustrated in Fig. SI. 1. The results shown below illustrate the maturation towards the stable speed for 10 % polypropylene, 76 % camphene, and 14 % camphor mixture. Three independent experiments were done. The observed speeds as functions of time are plotted in Fig. SI. 1 using different colors. The results for other compositions of the material showed a similar time of speed stabilization. Therefore, we neglected the initial 300 s of the pill motion for the results presented in the following subsections.

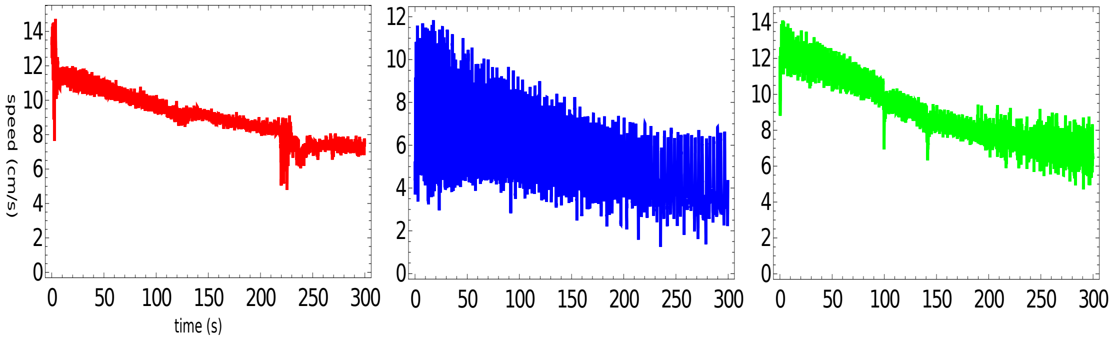

Figure SI. 1: The speed of a 4mm pill made of 10 % polypropylene, 76 % camphene and 14 % camphor mixture as a function of time for the initial stage of the pill motion. The motion was observed in a Petri dish with 11 cm diameter and for 1 cm water level.

SI.1.1 A pill made of 5 % polypropylene, 79.8 % camphene and 15.2 % camphor

We present the results of three independent experiments in which the pill motion was observed. The dish diameter was 11 cm. The trajectories and the distances between the pill center and the dish center are shown in Fig. SI. 2. These results were obtained by analysis of individual frames recorded at a rate of 30 fps. In each experiment, we observed a different character of motion. The red trajectory covers the central part of the dish fully. In the second experiment (the blue curve), we observed the rotational motion of the pill along the dish edge. The third experiment also showed such rotational motion, but it was interrupted by time intervals in which a random motion at a distance from the dish center was observed. Let us notice that in all cases, the pill never comes into contact with the dish edge. The largest recorded distance between the pill center and the dish center were 4.56, 4.46, and 4.91 cm; thus, the distance between the pill edge and the dish wall was almost always larger than 4 mm. Figure SI. 3 shows the radial distribution function $g(r)$ calculated from the distances seen of all frames of the recorded movie. Three curves illustrate functions

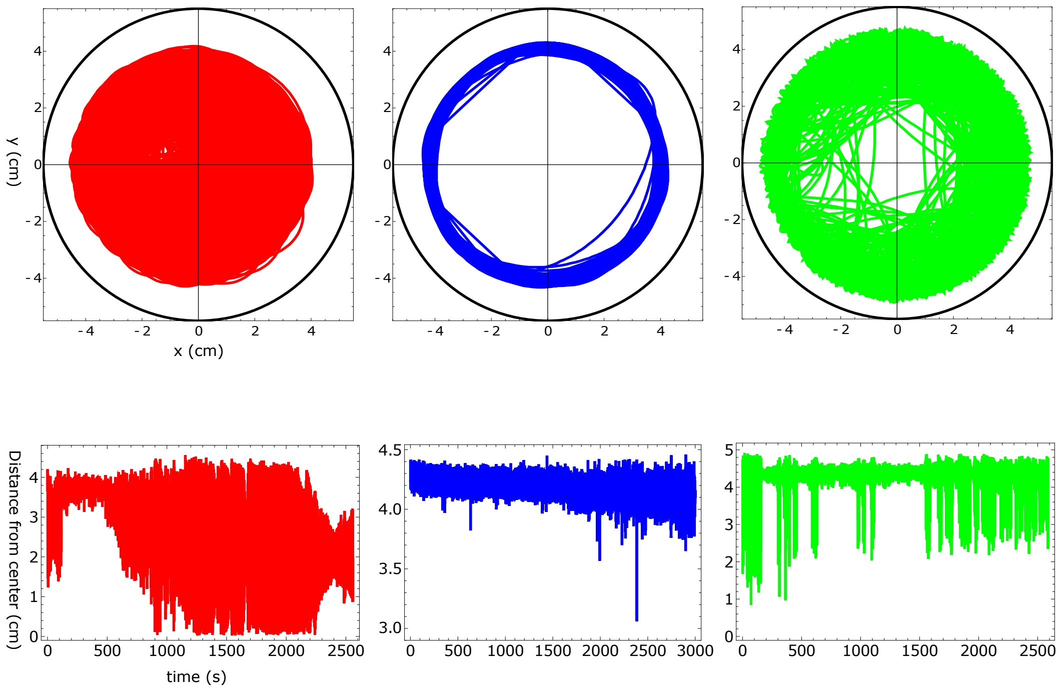

Figure SI. 2: The upper three figures show the trajectories of a 4mm pill made of 5 % polypropylene, 79.8 % camphene and 15.2 % camphor in a Petri dish with a 11 cm diameter. The black circle indicates the location of the dish edge. The distance between the pill center and the dish center as a function of time is plotted in figures located below the corresponding trajectories.

$g(r)$ measured in separate experiments. Figure SI. 4 presents the speed of a pill as a function of time. In all experiments, we observed an initial decrease of speed, but it does not exceed 25 % of the steady-state speed value. As expected, the most stable speed is observed for the most regular trajectory (rotation along the dish edge). Figure SI. 5 shows the probability distribution of speeds. The large figure shows the results of individual experiments. The distribution of speed values observed in all experiments is shown in the insert. The average values of speed observed for a pill made of 5 % polypropylene, 79.8 % camphene, and 15.2 % camphor were 4.81, 5.21 ,and 3.82 cm/s, .

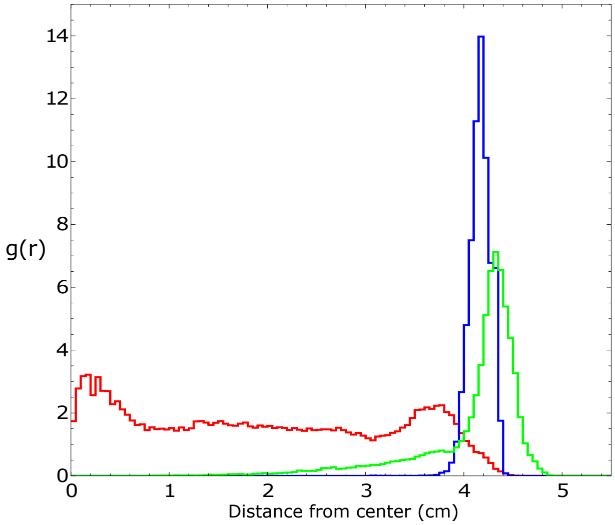

Figure SI. 3: The radial distribution function of the positions of pill centers calculated from the distances illustrated in Fig. SI. 2. The same color is used to draw the radial distribution function and the trajectory.

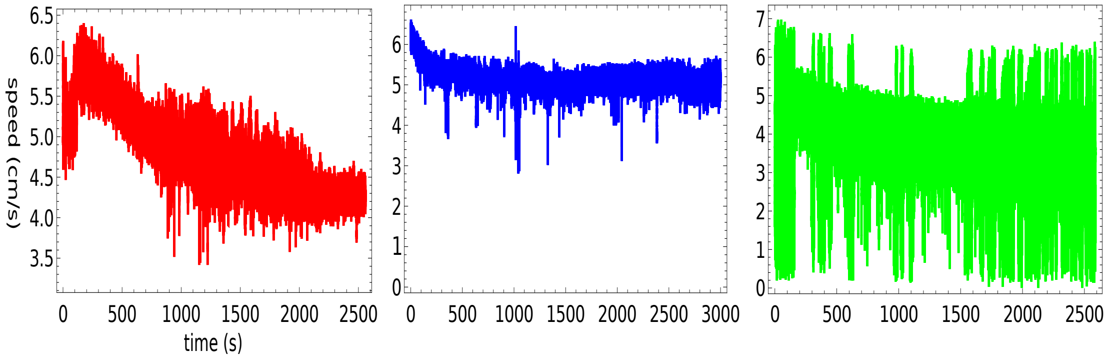

Figure SI. 4: The speed of a pill made of 5 % polypropylene, 79.8 % camphene and 15.2 % camphor as a function of time, as observed in three independent experiments.

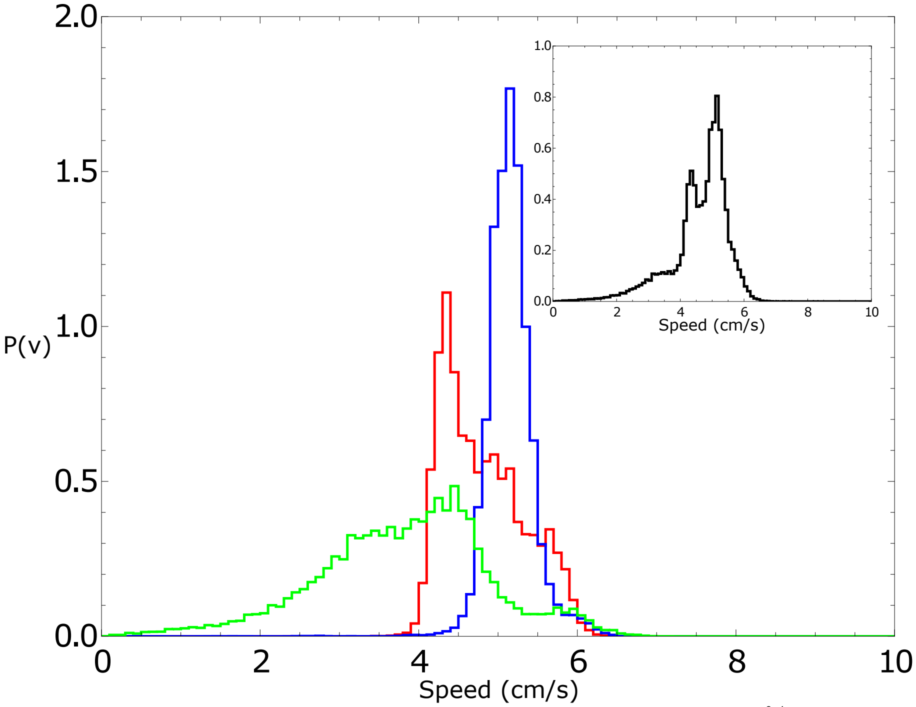

Figure SI. 5: The probability distribution of speeds for a pill made of 5 % polypropylene, 79.8 % camphene and 15.2 % camphor. The large figure shows results of individual experiments. The distribution of speed values observed in all experiments is shown in the insert.

SI.1.2 A pill made of 10 % polypropylene, 76 % camphene and 14 % camphor

The dish diameter was 12 cm. The trajectories and the distances between the pill center and the dish center are shown in Fig. SI. 6. These results were obtained by analysis of individual frames recorded at a rate of 25 fps. The red trajectory covers the central part of the dish fully. In the second and the third experiments (blue and green curves), we observed the rotational motion of the pill along the dish edge. The pill does not come into contact with the dish edge in any of the experiments. The largest recorded distances between the pill center and the dish center were 5.19, 5.25, and 5.13 cm, respectively; thus, the distance between the pill edge and the dish wall was always larger than 5 mm. Figure SI. 7 shows the radial distribution function $g(r)$ calculated from the distances seen of all frames of the recorded movie. The curves present $g(r)$ measured in separated experiments.

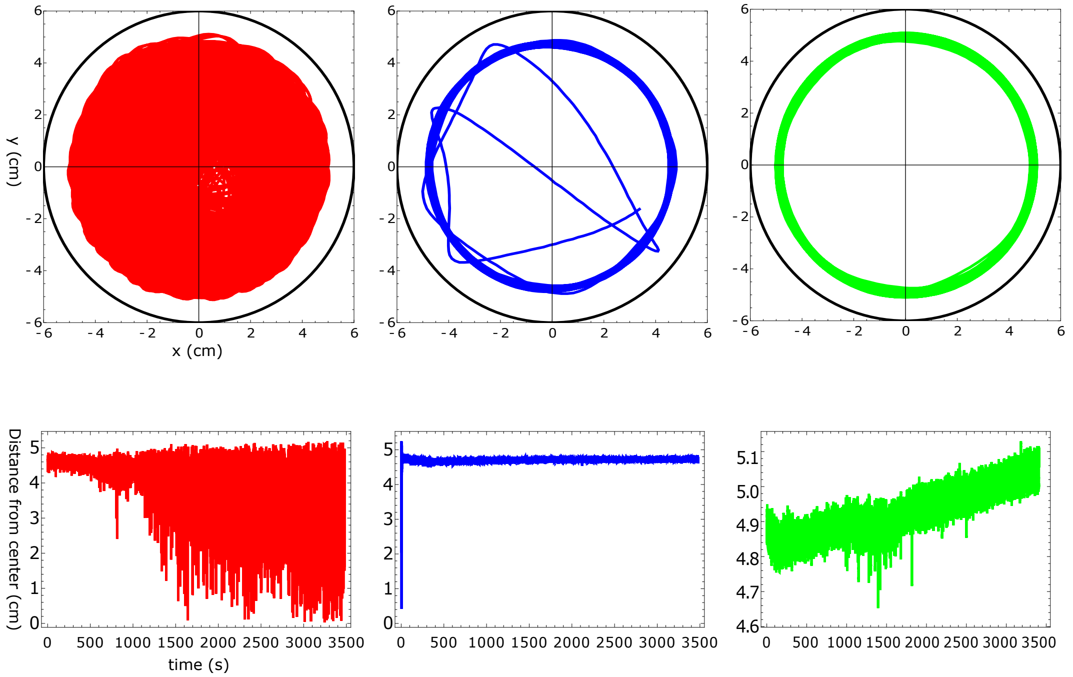

Figure SI. 6: The upper three figures show the trajectories of a 4mm pill made of 10 % polypropylene, 79.8 % camphene, and 15.2 % camphor in a Petri dish with a 12 cm diameter. The black circle indicates the location of the dish edge. The distances between the pill center and the dish center observed in individual experiments as functions of time are plotted in figures located below the corresponding trajectories.

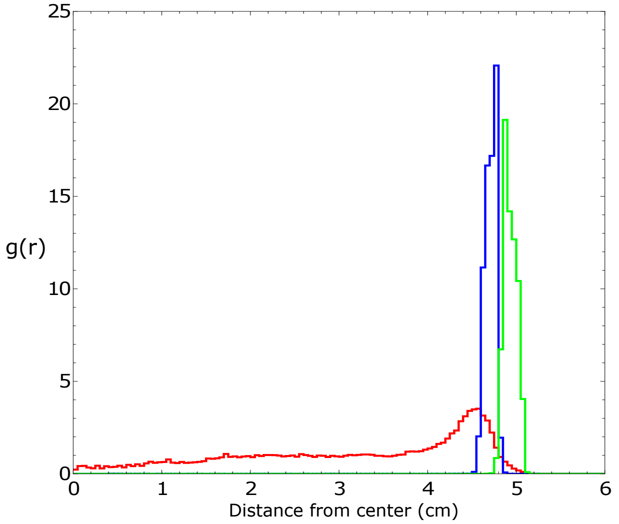

Figure SI. 7: The radial distribution function of the positions of pill centers calculated from the distances illustrated in Fig. SI. 6. The same color is used to draw the radial distribution function and the trajectory.

Figure SI. 8 presents the speed of a pill as a function of time. In all experiments we observed an initial decrease of speed but it does not exceed 25 % of the steady-state speed value. As expected, the speed is most stable for the most regluar trajectory (rotation along the dish edge). Figure SI. 9 shows the probability distribution of speeds. The large figure shows results of individual experiments. The distribution of speed values observed in all experiments is shown in the insert. The average values of speed observed for a pill made of 10 % polypropylene, 76 % camphene and 14 % camphor were 4.73, 5.32 ,and 5.53 cm/s .

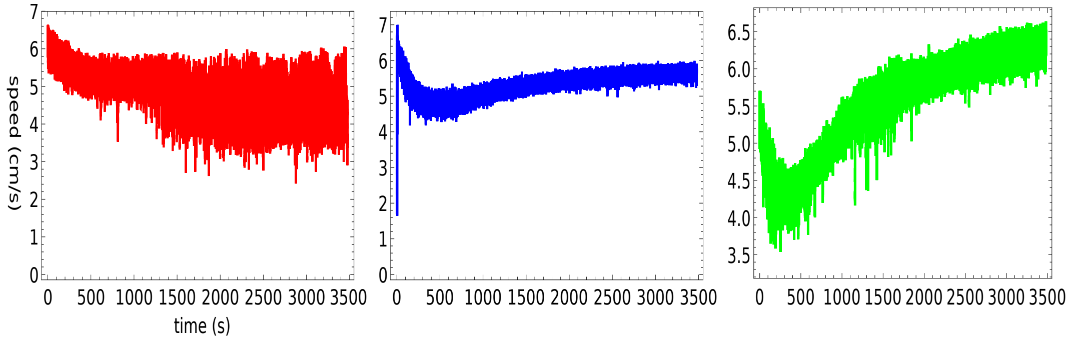

Figure SI. 8: The speed of a pill made of 10 % polypropylene, 76 % camphene and 14 % camphor as a function of time, as observed in three independent experiments.

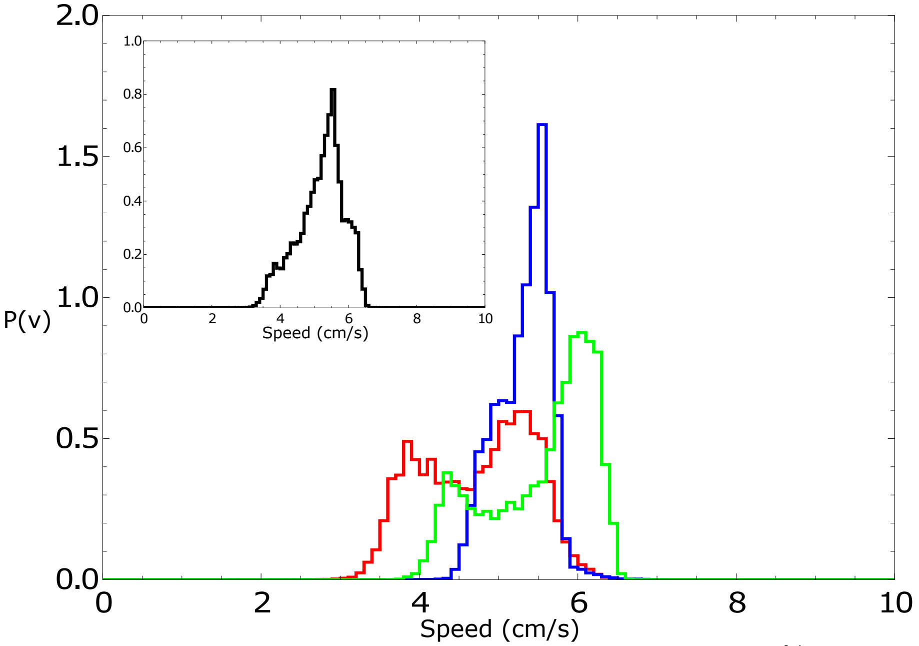

Figure SI. 9: The probability distribution of speeds of pill made of 10 % polypropylene, 76 % camphene and 14 % camphor. The large figure shows results of individual experiments. The distribution of speed values observed in all experiments is shown in the insert.

SI.1.3 A pill made of 5 % polypropylene, 63.7 % camphene and 31.3 % camphor

The dish diameter was 11 cm. The trajectories and the distances between the pill center and the dish center are shown in Fig. SI. 10. These results were obtained by analysis of individual frames recorded at a rate of 30 fps. In the first and second experiments (the red and blue curves), we observed the rotational motion of the pill along the dish edge. The third experiment also showed rotational motion, but the trajectory covered a large part of the dish area. Let us notice that there is no contact between pill and dish edge in any of the experiments. The largest recorded distances between the pill center and the dish center were 4.44, 4.53, and 4.71 cm, respectively; thus, the distance between the pill edge and the dish wall was always larger than 5 mm. Figure SI. 11 shows the radial distribution function $g(r)$ calculated from the distances seen of all frames of the recorded movie. The curves illustrate $g(r)$ measured in separated experiments.

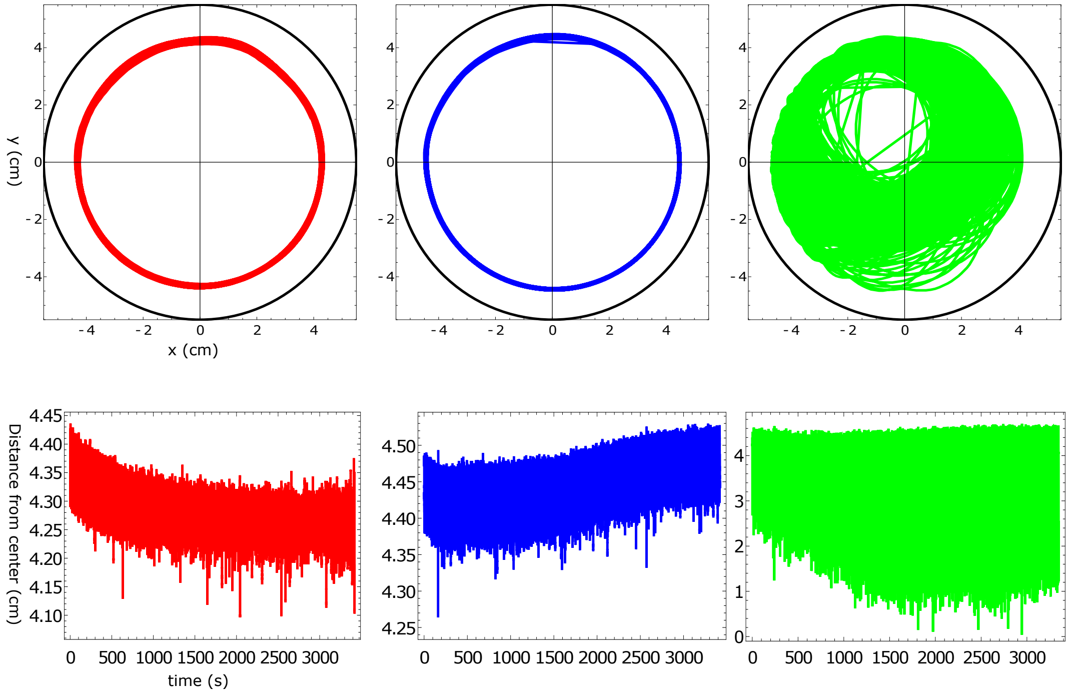

Figure SI. 10: The upper three figures show the trajectories of a 4mm pill made of 5 % polypropylene, 63.7 % camphene, and 31.3 % camphor in a Petri dish with an 11 cm diameter. The black circle indicates the location of the dish edge. The distances between the pill center and the dish center observed in individual experiments as functions of time are plotted in figures located below the corresponding trajectories.

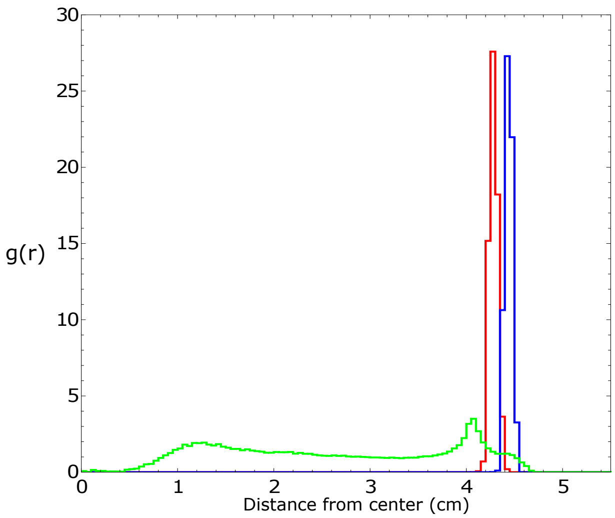

Figure SI. 11: The radial distribution function of the positions of pill centers calculated from the distances illustrated in Fig. SI. 10. The same color is used to draw the radial distribution function and the trajectory.

Figure SI. 12 presents the speed of a pill as a function of time. In all experiments we observed an initial decrease of speed but it does not exceed 30 % of the steady-state speed

value. As expected speed is the most stable for the most regular trajectory (rotation along the dish edge). Figure SI. 13 shows the probability distribution of speeds. The large figure shows results of individual experiments. The distribution of speed values observed in all experiments is shown in the insert. The average value of speed observed for a pill made of 5 % polypropylene, 63.7 % camphene and 31.3 % camphor were 5.09, 6.60 ,and 3.93 cm/s .

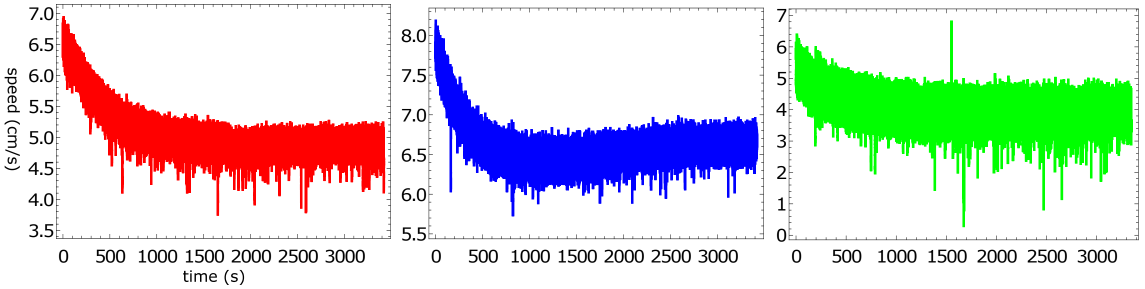

Figure SI. 12: The speed of a pill made of 5 % polypropylene, 79.8 % camphene and 15.2 % camphor as a function of time, as observed in three independent experiments.

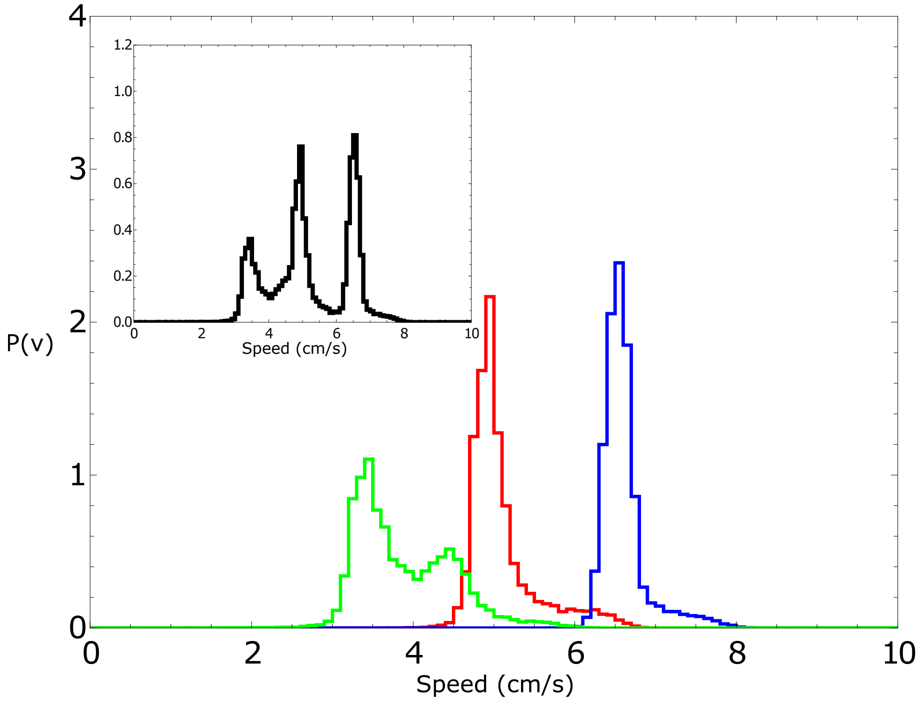

Figure SI. 13: The probability distribution of speeds. The large figure shows results of individual experiments. The distribution of speed values observed in all experiments is shown in the insert.

SI.1.4 A pill made of 10 % polypropylene, 60 % camphene and 30 % camphor

The dish diameter was 12 cm. The trajectories and the distances between the pill center and the dish center are shown in Fig. SI. 14. These results were obtained by analysis of individual

frames recorded at a rate of 25 fps. In each experiment, we observed a different character of motion. In the first and second experiments (the red and blue curves), we observed a complex motion covering the whole surface of the dish. In the second experiment (the blue curve), this complex motion switches into the rotational motion along the dish edge after 30 minutes. In the third experiment (the green curve), we also observed the rotational motion of the pill along the dish edge. The dispersion of the radii decreased with time. Let us notice that there is no contact between pill and dish edge in any of the experiments. The largest recorded distances between the pill center and the dish center were 5.41, 5.15, and 5.10 cm, respectively; thus, the distance between the pill edge and the dish wall was almost always larger than 4 mm. Figure SI. 15 shows the radial distribution function $g(r)$ calculated from the distances seen of all frames of the recorded movie. The curves show $g(r)$ measured in separate experiments.

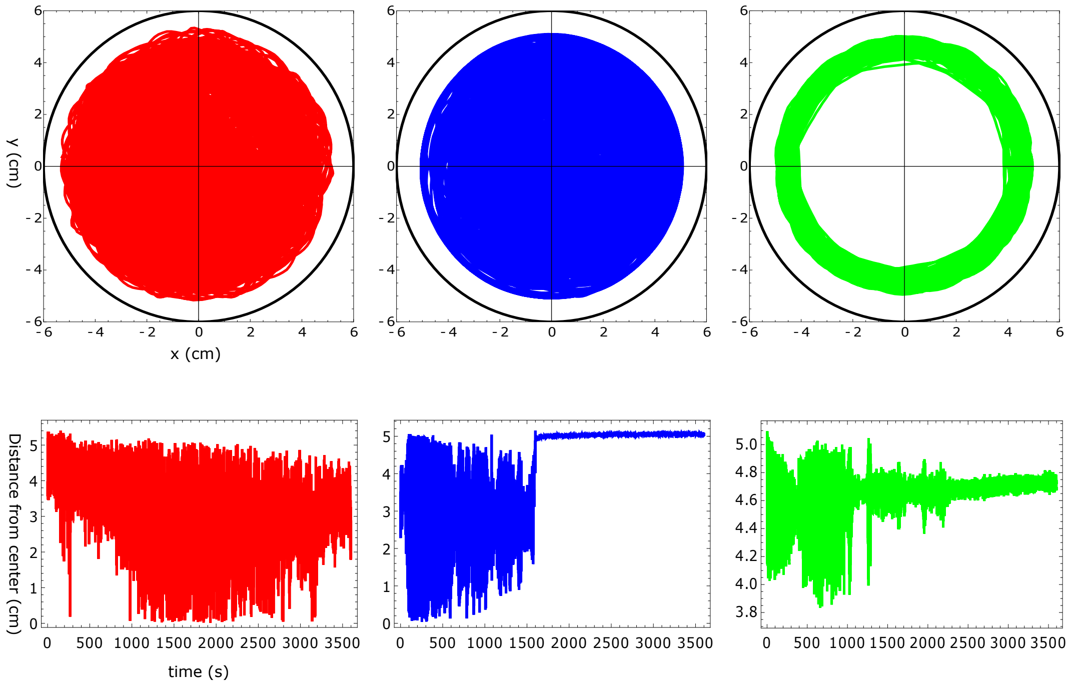

Figure SI. 14: The upper three figures show the trajectories of a 4mm pill in a Petri dish with a 12 cm diameter. The black circle indicates the location of the dish edge. The distances between the pill center and the dish center observed in individual experiments as functions of time are plotted in figures located below the corresponding trajectories.

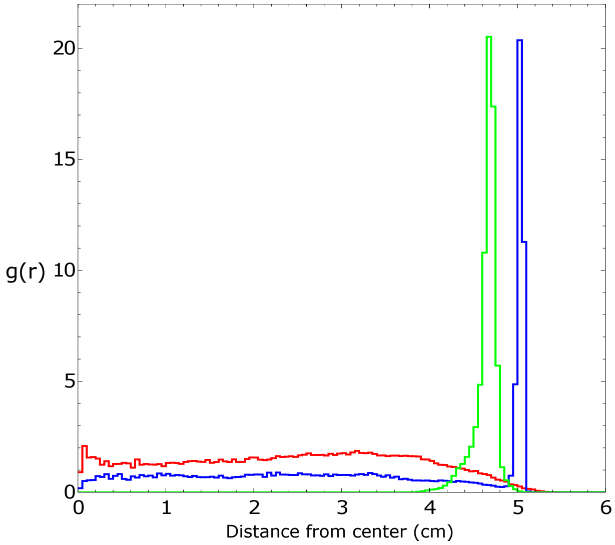

Figure SI. 15: The radial distribution function of the positions of pill centers calculated from the distances illustrated in Fig. SI. 14. The same color is used to draw the radial distribution function and the trajectory.

Figure SI. 16 presents the speed of a pill as a function of time. In all experiments, we observed an initial decrease of speed, but it does not exceed 25 % of the steady-state speed value. As expected, speed is most stable for the most regular trajectory (rotation along the dish edge). Figure SI. 17 shows the probability distribution of speeds. The large figure shows the results of individual experiments. The distribution of speed values observed in all experiments is shown in the insert. The average values of speed observed for a pill made of 10 % polypropylene, 60 % camphene, and 30 % camphor were 3.01, 5.30 ,and 5.90 cm/s in separate experiments.

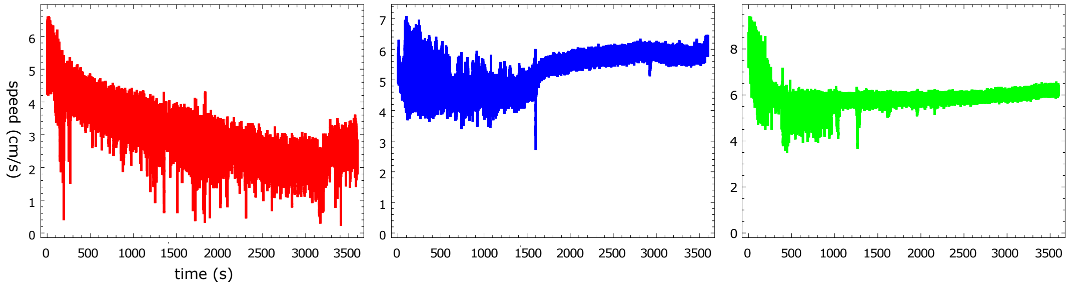

Figure SI. 16: The speed of a pill made of 10 % polypropylene, 60 % camphene and 30 % camphor as a function of time, as observed in three independent experiments.

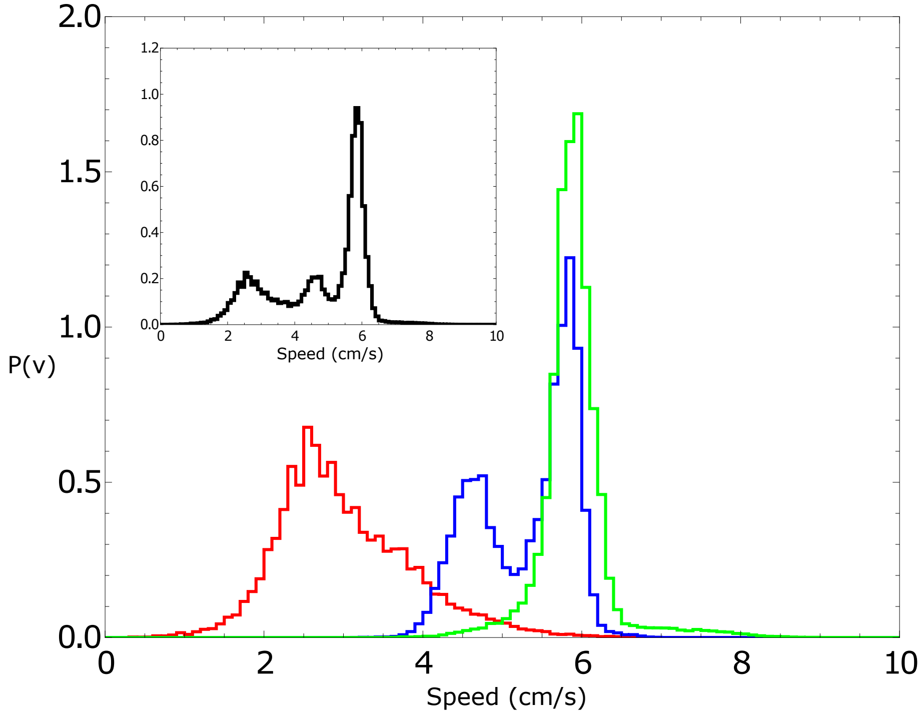

Figure SI. 17: The probability distribution of speeds. The large figure shows results of individual experiments. The distribution of speed values observed in all experiments is shown in the insert.

SI.1.5 A pill made of 5 % polypropylene, 47.5 % camphene and 47.5 % camphor

The dish diameter was 11 cm. The trajectories and the distances between the pill center and the dish center are shown in Fig. SI. 18. These results were obtained by analysis of individual frames recorded at a rate of 30 fps. In each experiment, we observed a different character of motion. The red trajectory shows the motion around the dish center with a large dispersion of radii that, after 2200 seconds, stabilizes to rotation characterized by a constant radius. In the second experiment (the blue curve), we observed the rotational motion of the pill along the dish edge with a fixed radius. The third experiment also showed complex motion that mainly occurs at a 1 cm distance from the dish center. In none of the cases, the pill comes into contact with the dish edge. The largest recorded distance between the pill center and the dish center were 4.78, 4.32, and 4.66 cm, respectively; thus, the distance between the pill edge and the dish wall was always larger than 5 mm. Figure SI. 19 shows the radial distribution function $g(r)$ calculated from the distances seen of all frames of the recorded movie. The separated curves show $g(r)$ measured in separated experiments.

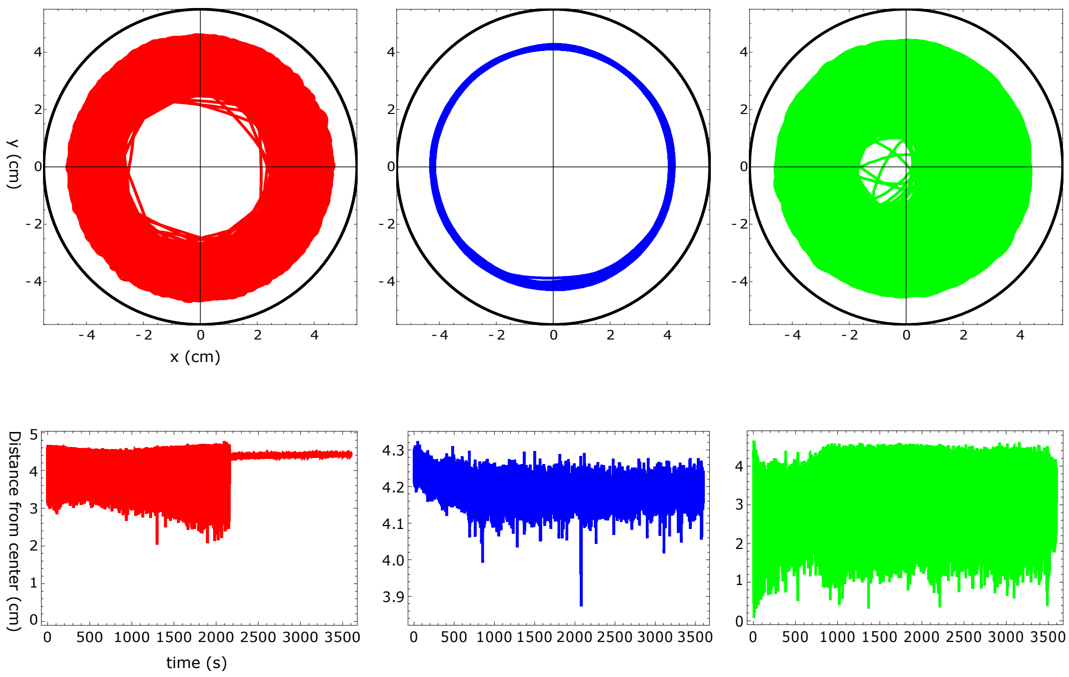

Figure SI. 18: The upper three figures show the trajectories of a 4mm pill made of 5 % polypropylene,47.5 % camphene and 47.5 % camphor in a Petri dish with the 11 cm diameter. The black circle indicates the location of the dish edge. The distances between the pill center and the dish center observed in individual experiments as functions of time are plotted in figures located below the corresponding trajectories.

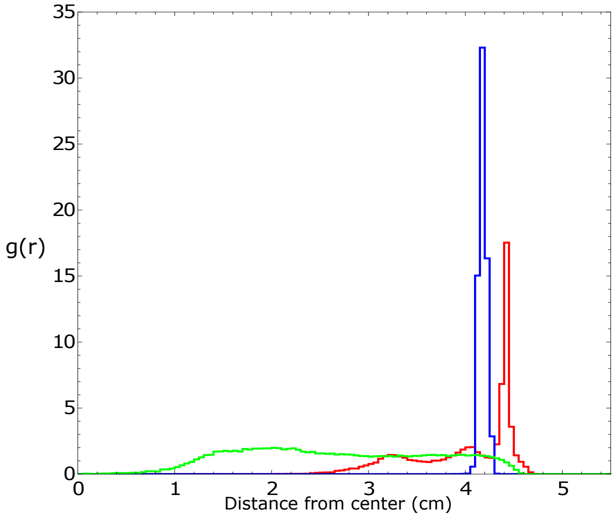

Figure SI. 19: The radial distribution function of the positions of pill centers calculated from the distances illustrated in Fig. SI. 18. The same color is used to draw the radial distribution function and the trajectory.

Figure SI. 20 presents the speed of the pill as a function of time. In all experiments, we observed an initial decrease of speed, but it does not exceed 25 % of the steady-state speed

value. As expected, the speed is most stable for the most regular trajectory (rotation along the dish edge). Figure SI. 21 shows the probability distribution of speeds. The large figure presents the results of individual experiments. The distribution of speed values observed in all experiments is shown in the insert. The average value of speed observed for a pill made of 5 % polypropylene, 47.5 % camphene, and 47.5 % camphor were 5.23, 3.99 ,and 4.57 cm/s .

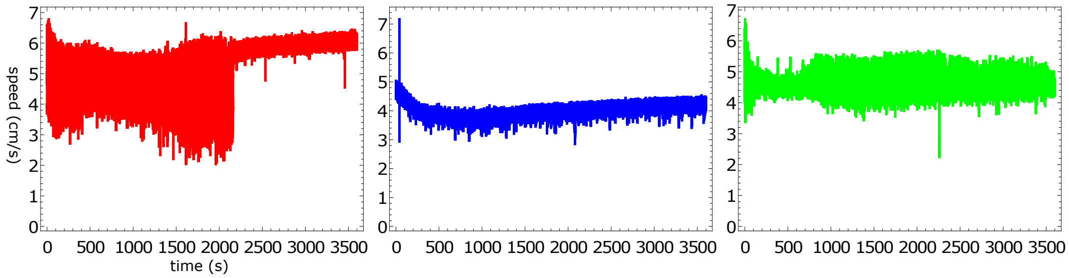

Figure SI. 20: The speed of a pill made of 5 % polypropylene, 47.5 % camphene and 47.5 % camphor as a function of time, as observed in three independent experiments.

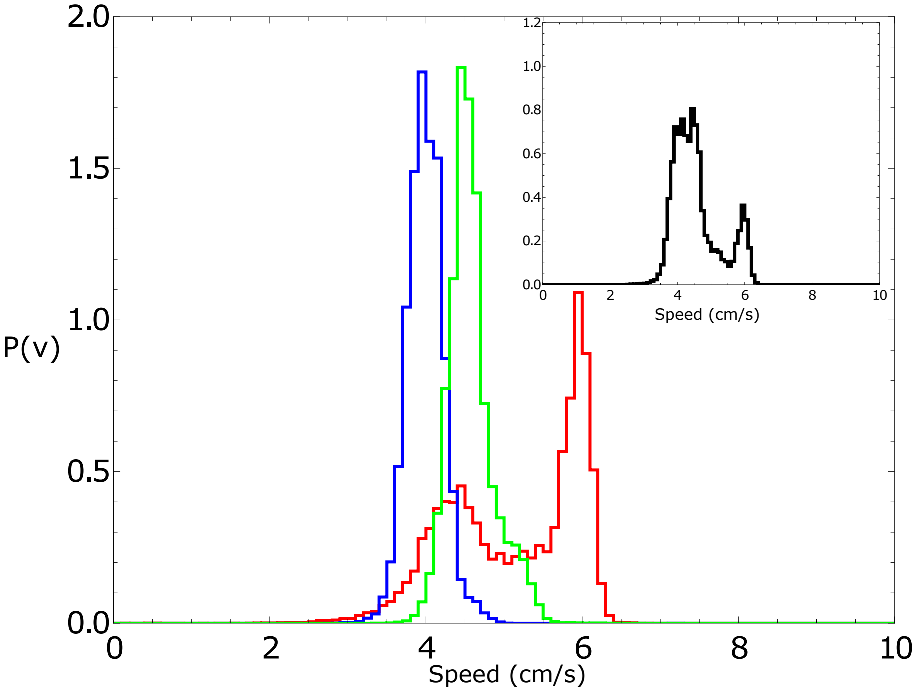

Figure SI. 21: The probability distribution of speeds. The large figure hows results of individual experiments. The distribution of speed values observed in all experiments is shown in the insert.

SI.1.6 A pill made of 10 % polypropylene, 45 % camphene and 45 % camphor

The results presented in this subsection are also presented in the paper as the illustration of the properties of camphene-camphor-polypropylene plastic.

The dish diameter was 12 cm. The trajectories and the distances between the pill center and the dish center are shown in Fig. SI. 22. These results were obtained by analysis of individual frames recorded at a rate of 25 fps. In each experiment, we observed a different character of motion. The red trajectory covers the central part of the dish. In the second experiment (the blue curve), we observed the rotational motion of the pill along the dish edge. The third experiment also showed such rotational motion, but it was separated by time intervals in which a complex motion at a distance from the dish center was observed. Let us notice that the pill never comes to contact with the dish edge in any of the experiments. The largest recorded distance between the pill center and the dish center were 5.62, 5.23, and 5.41 cm, respectively; thus, the distance between the pill edge and the dish wall was almost always larger than 2 mm. Figure SI. 23 shows the radial distribution function $g(r)$ calculated from the distances seen of all frames of the recorded movie. Curves illustrate functions $g(r)$ measured in separate experiments.

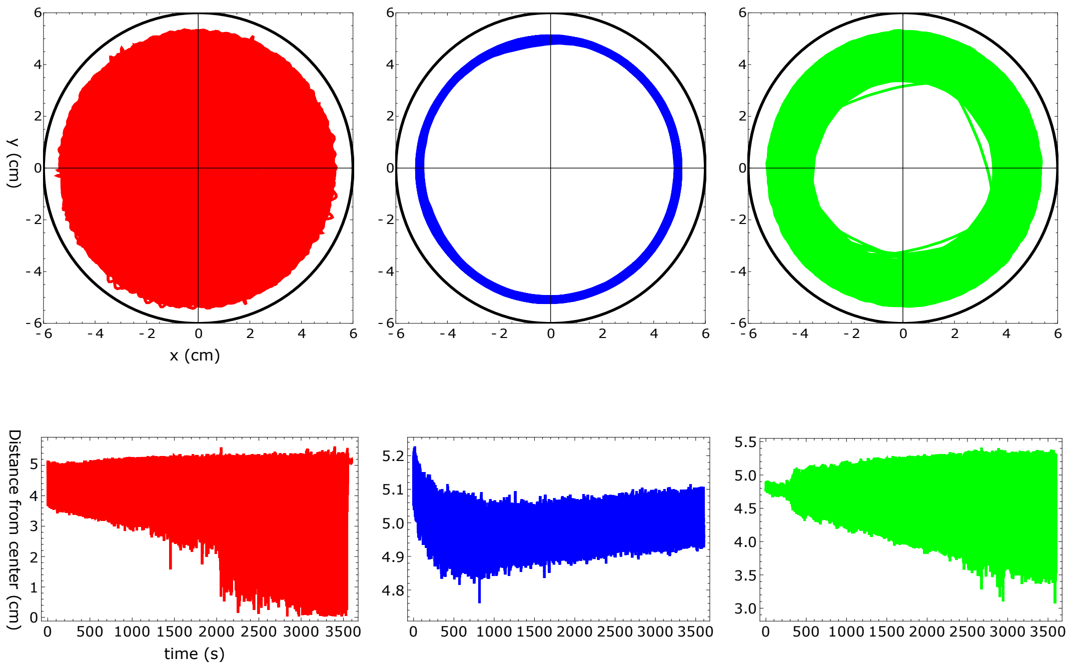

Figure SI. 22: The upper three figures show the trajectories of a 4mm pill made of 10 % polypropylene, 45 % camphene and 45 % camphor in a Petri dish with a 12 cm diameter. The black circle indicates the location of the dish edge. The distances between the pill center and the dish center observed in individual experiments as functions of time are plotted in figures located below the corresponding trajectories.

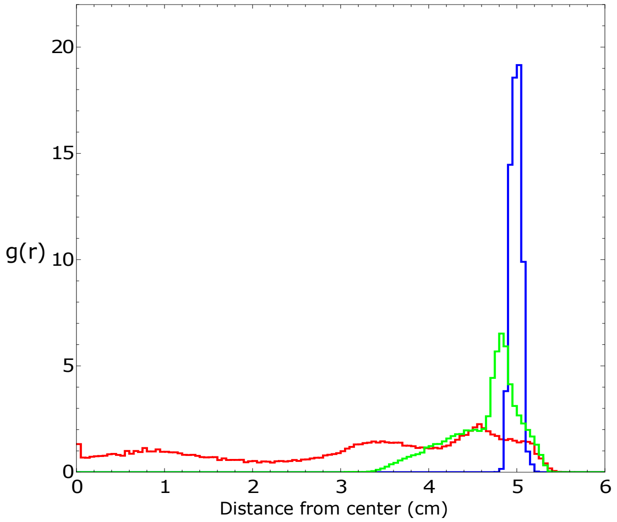

Figure SI. 23: The radial distribution function of the positions of pill centers calculated from the distances illustrated in Fig. SI. 22. The same color is used to draw the radial distribution function and the trajectory.

Figure SI. 24 presents the speed of a pill made of 10 % polypropylene, 45 % camphene and 45 % camphor as a function of time. In all experiments, we observed an initial decrease of speed, but it does not exceed 40 % of the steady-state speed value. As expected, speed is the most stable for the most regular trajectory (rotation along the dish edge). Figure SI. 25 shows the probability distribution of speeds. The large figure shows the results of individual experiments. The distribution of speed values observed in all experiments is shown in the insert. The average value of speed observed for a pill made of 10 % polypropylene, 45 % camphene and 45 % camphor were 5.28, 7.63 ,and 7.57 cm/s .

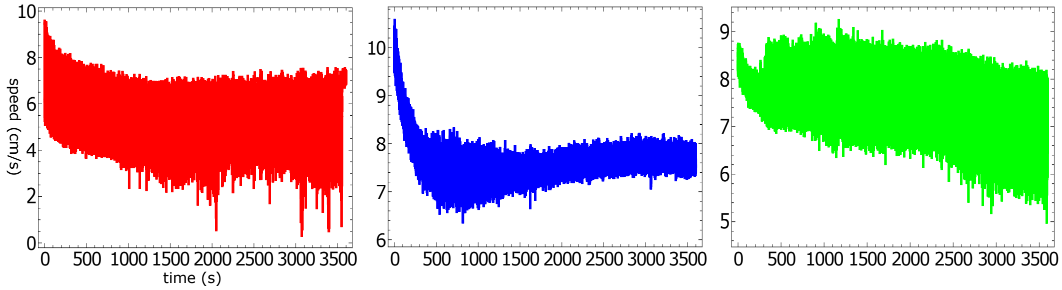

Figure SI. 24: The speed of a pill made of 10 % polypropylene, 45 % camphene and 45 % camphor as a function of time, as observed in three independent experiments.

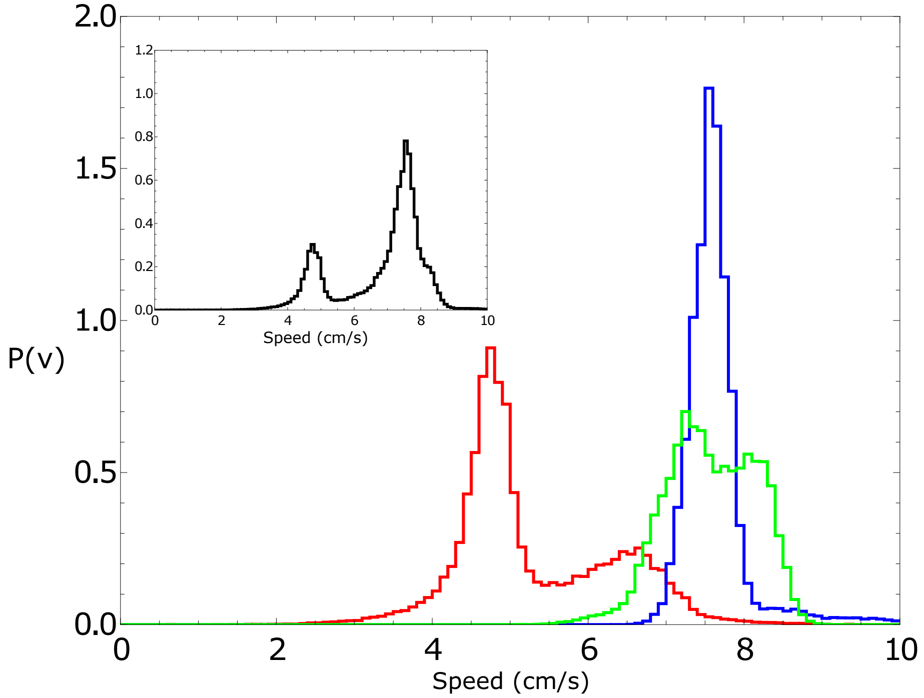

Figure SI. 25: The probability distribution of speeds. The large figure shows results of individual experiments. The distribution of speed values observed in all experiments is shown in the insert.
